# Adult mental health outcomes of adolescent depression and co-occurring alcohol use disorder: a longitudinal cohort study

**DOI:** 10.1007/s00787-024-02596-3

**Published:** 2024-10-29

**Authors:** Hannes Bohman, Sara Brolin Låftman, Iman Alaie, Richard Ssegonja, Ulf Jonsson

**Affiliations:** 1 Uppsala University, Uppsala, Sweden; 2 Karolinska Institutet, Stockholm, Sweden; 3 Stockholm University, Stockholm, Sweden

**Keywords:** Adolescent depression, Alcohol, Cohort study, Comorbidity, Epidemiology, Longitudinal design, Substance use

## Abstract

Depression and alcohol use disorder (AUD) are frequently co-occurring in adolescence, which often goes undetected in routine care. While this may potentially compromise treatment effectiveness and lead to a less favourable long-term prognosis, few longitudinal studies have followed this group into adulthood. The aim of this study was to explore the risk for adult depression, anxiety disorders, suicidality, and AUD in adolescents with concurrent depression and AUD. The study was based on the Uppsala Longitudinal Adolescent Depression Study (ULADS), a Swedish prospective cohort study. Diagnostic interviews were conducted in adolescence (age 16-17) and adulthood (around age 30). Adolescents with concurrent depression and AUD (n = 38) were compared with peers having only depression (n = 189) or neither of the conditions (n = 144). Logistic regression was used to calculate adjusted odds ratios (aORs) with 95% confidence intervals (CIs). Adolescents with concurrent depression and AUD were more likely than their non-affected peers to experience adult depressive episodes (aOR, 5.33; 95% CI, 2.22–12.83), anxiety disorders (4.05; 1.77–9.27), suicidality (5.37; 2.28–12.66), and AUD (7.68; 2.59–22.81). Notably, 34% of adolescents with both depression and AUD subsequently experienced both these conditions as adults, compared to 7% of adolescents with only depression. Adolescents suffering only from depression were less likely than those with both conditions to experience suicidality (0.44; 0.21–0.95) and AUD in adulthood (0.18; 0.07–0.44). These findings underscore the clinical imperative to identify adolescents with this comorbidity. Recognition of the poor long-term prognosis can inform targeted interventions for this vulnerable group, ultimately improving health and well-being throughout the life course.

**Supplementary Information:**

The online version contains supplementary material available at 10.1007/s00787-024-02596-3.

## Introduction

Depression assumes a substantive role in the global burden of disease among adolescents [1], with an estimated global point prevalence of 8% for major depressive disorder and 34% for elevated depressive symptoms [2]. Depression is frequently co-occurring with other psychiatric conditions such as anxiety, conduct disorder, and substance misuse, including alcohol dependency and/or abuse [3]. The ramifications of adolescent depression extend well beyond the immediate timeframe, exerting enduring adverse effects of significant magnitude. Of notable concern is the elevated risk of future relapse or persistence extending into adulthood, with an estimated 60% of individuals who experienced depression in adolescence also suffering from recurrence between 19 and 30 years of age [4]. Furthermore, the presence of adolescent depression amplifies the vulnerability to other subsequent mental disorders [5], as well as a range of adverse functional and psychosocial outcomes [6], thereby compounding the burden of illness faced by affected individuals.

Alcohol use disorder (AUD) represents a prevalent issue among adolescents within the Swedish population. Population-based data from 2023 reveal that 70% of upper secondary school students in Sweden have consumed alcohol during the past year, and approximately 20% engaged in heavy episodic drinking [7]. According to a recent Swedish study, approximately one-third of alcohol consumers aged 17-18 met the criteria for an AUD [8]. It is important to recognise that AUD exerts detrimental effects on various aspects of a young individual’s life, including academic performance and interpersonal relationships with peers and family members, and imposes significant social and health-related costs [9]. Genetically informative research supports the notion of a ‘self-medication’ hypothesis, as the risk of developing adult alcohol misuse was found to be substantially increased by a prior episode of depression, also when accounting for potential confounding by familial factors [10]. However, the association between alcohol problems and depression has been shown to be reciprocal [11]. The co-occurrence of adolescent depression and AUD is well-established, with clinical evidence suggesting that dual diagnosis (i.e., the co-occurrence of both conditions) is present in a substantial proportion of adolescents with AUD, ranging from 50% to 80% in clinical samples [12]. It has been estimated that approximately 2% of teenagers experience concurrent AUD and depression [13]. This high level of comorbidity raises concerns regarding the potential challenges in effectively treating both conditions in such cases.

Existing research focusing on young individuals with a dual diagnosis of depression and AUD consistently highlights their diminished treatment adherence and inferior treatment outcomes when compared to those with a singular diagnosis [14, 15]. Undisclosed alcohol abuse and dependency issues may also go unnoticed in adolescents being treated for other mental health conditions [16, 17]. This scenario may not only compromise the efficacy of treatment for the depressive disorder but also result in failures to provide adequate treatment for AUD.

Previous research indicates that the presence of a dual diagnosis may amplify the risk of suicidal behaviour, surpassing the risks associated with each condition in isolation [15]. Existing evidence supports the notion that both depression [18] and AUD [19] engender persistent psychosocial challenges throughout young adulthood. Importantly, it is plausible that both adolescent depression and AUD independently may exert an adverse influence on the developing brain [20]. Consequently, a dual diagnosis may give rise to a synergistic interplay, exacerbating the detrimental impact on brain functioning and potentially impeding an individual’s ability to achieve optimal functioning well into adulthood.

Given the multifaceted challenges associated with the dual diagnosis of depression and AUD during adolescence, it is reasonable to infer that individuals in this group face an especially unfavourable prognosis regarding their potential for a healthy and functional adult life. Presently, our understanding of the connections between co-occurring depression and AUD in adolescence and the subsequent long-term risk of mental illness and addiction remains limited [21, 22]. Using a New Zealand birth cohort, Boden & Foulds [23] found that the co-existence of adolescent depression and AUD did not significantly predict major depression in early adulthood when controlling for certain confounding factors. They concluded that comorbidity during adolescence was more likely a risk marker rather than a causal factor for poor outcomes in adulthood, challenging the notion that specific treatments targeting comorbid condition in adolescence would improve long-term outcomes.

A wide range of co-occurring factors may contribute to a more unfavourable long-term prognosis of depression (e.g., male sex, psychological trauma, low socioeconomic status, ADHD, disruptive behavioural problems, interpersonal conflicts within the family, and parental depression) [23]. However, it is not known whether co-occurring AUD increases the risk of other mental health outcomes in adulthood. Gaining deeper insights into these longitudinal associations could be vital to the development of early prevention and treatment targets and, thereby, more effective use of limited resources in child and adolescent mental health services.

This study sought to expand on the available literature on co-occurring depression and AUD in adolescence by exploring a wider range of common mental health outcomes in the longer term. The primary objective was to assess whether this particular group exhibits heightened risks of depression, anxiety disorders, suicidality, and AUD in early adulthood. The following research questions guided our investigation:

1. Is the long-term mental health prognosis for adolescents with depression and AUD poorer compared with adolescents without either of these conditions?
2. Does the presence of concurrent AUD and depression in adolescence exacerbate the long-term mental health prognosis for adolescents with depression, in comparison to those affected by depression or AUD alone?
3. Do the associations persist after controlling for sex and other underlying factors present in childhood and adolescence, such as trauma (physical or sexual violence), socioeconomic status, ADHD, oppositional defiant disorder, conduct disorder, interpersonal conflicts within the family, and parental depression?

## Methods

### Design

The work utilised a prospective epidemiological cohort study, Uppsala Longitudinal Adolescent Depression Study (ULADS) [3]. The study was initiated in Uppsala, Sweden, during 1991–1992, with the primary objective to examine the prevalence of depression in adolescents employing a case-control design. First-year students starting upper-secondary school in Uppsala, Sweden, were screened for depression using self-rated questionnaires. Adolescents with a screen-positive profile and peers matched for sex, age, and school year/class were invited for a diagnostic interview. Participants were thereafter followed up through interviews approximately 15 years later. The cohort has been profiled in detail elsewhere [3]. The original study was approved by the Ethical Committee of Uppsala University and subsequent phases of the study were approved by the Regional Ethical Review Board in Uppsala, Sweden (03-253; 2006/024/1–3; 2015/449/1–2). All participants gave their informed consent prior to inclusion.

### Procedure and participants

The baseline screening comprised students in their first year of upper secondary school (aged 16–17 years) and those within the same age group who had discontinued their education. Out of 2465 eligible individuals, 2300 (93%) willingly took part by responding to two self-report questionnaires: The Beck Depression Inventory-Child, BDI [24], and the Center for Epidemiological Studies-Depression Scale for Children, CES-DC [25]. Adolescents scoring high on either scale (BDI-C≥16 or CES-DC≥30), or reporting a suicide attempt, underwent diagnostic interviews using the Diagnostic Interview for Children and Adolescents, DICA-R-A, which assesses both current and lifetime disorders [26]. A total of 301 of 340 invited adolescents completed the interview (19 refused and 20 had left school), with no significant difference in mean score on the BDI or CES-DC between those who completed and those who did not. A control group including individuals with low depression scores (BDI-C<16 and CES-DC<30), matched for sex, age, and school class underwent the same assessment procedure. Among controls, 37 refused or could not be reached. Their mean scores in the screening did not differ significantly from those interviewed. A total of 631 adolescents participated in the interview and were subsequently invited to a follow-up assessment between 30 and 33 years of age. This follow-up assessment included a structured diagnostic interview using the Mini International Neuropsychiatric Interview (M.I.N.I.) Plus [27], assessing current and previous conditions from age 19 onward. In addition, self-reported information on ongoing hazardous use of alcohol and drugs was gathered at this stage through the Alcohol Use Disorders Identification Test (AUDIT) [28] and the Drug Use Disorders Identification Test (DUDIT) [29], respectively. A total of 409 individuals participated in the follow-up assessment, yielding a response rate of 65%. The present study included participants who took part in both waves of measurement. Participants with a history of a hypomanic episode in adolescence (n = 27) were excluded due to the distinct aetiology, trajectory, and clinical management of bipolar disorder. This resulted in an analytic study sample of 382 individuals.

### Study variables

#### Exposure

Mental health conditions in adolescence were ascertained using information from the DICA-R-A, the BDI and the CES-DC. Depression was operationalised as either a lifetime depressive disorder according to the DICA-R-A (n = 187) or subsyndromal depression defined as positive screening (n = 40). AUD was defined as either alcohol misuse or dependence. Based on the presence of depression and AUD, four distinct sub-groups were outlined:

- (1) no depression or AUD in adolescence (n = 144);
- (2) AUD but not depression in adolescence (n = 11);
- (3) depression but no AUD in adolescence (n = 189);
- (4) co-occurring depression and AUD in adolescence (n = 38).

#### Outcomes

The outcomes of interest encompassed mental health conditions in adulthood (major depression, anxiety disorder, suicidality, AUD, and drug abuse), ascertained using the M.I.N.I. [27], and current self-assessed hazardous drinking and drug abuse measured by AUDIT and DUDIT. Depression was defined as at least one major depressive episode since age 19. Anxiety disorder was defined as panic disorder since age 19, or any other anxiety disorder during the past 12 months (M.I.N.I.). AUD and drug abuse disorder during adulthood were defined as fulfilling the M.I.N.I. criteria at least once since age 19. Suicidality was evaluated based on non-mutually exclusive information concerning death wishes, suicidal thoughts, suicide plans, suicide attempts, and suicide attempts resulting in hospitalisation since age 19. Current hazardous drinking at follow-up was defined as an AUDIT score of ≥8 for men and ≥6 for women [30], and current hazardous drug use as a DUDIT score of >5.

#### Covariates

The covariates encompassed sex, as well as physical/sexual trauma, parental unemployment, reduced family income, and major conflicts within the family, assessed using self-report data obtained at baseline through the Life Events Form developed by Coddington [31]. Participants were asked whether each life event occurred within the past year or earlier in life. The responses were then combined to create binary measures indicating the presence or absence of each life event. ADHD/defiant disorder/conduct disorder and drug use at baseline were assessed by DICA-R-A [26]. Additionally, parental depression was assessed during the follow-up interview using a study-specific chart designed to capture various psychiatric diagnoses reported by the interviewee regarding their close relatives. The data were coded to indicate whether at least one parent had experienced depression.

### Statistical method

To assess differences in sample characteristics at baseline and follow-up, we used χ^2^ tests. Attrition from baseline to the follow-up assessment was analysed using a logistic regression model predicting participation in the follow-up interview based on baseline characteristics. Logistic regression models estimating odds ratios (ORs) with 95% confidence intervals (CIs) were performed to analyse the associations between exposures (depression and AUD in adolescence) and outcomes (depressive episodes, anxiety disorders, suicidality, and AUD in adulthood). Univariate analyses were initially conducted to examine crude associations of different combinations of depression and AUD during adolescence (AUD only, depression only, or both) with each of the outcomes in adulthood, with having neither of the conditions in adolescence as the reference category. Subsequently, multivariable models were employed to adjust for the complete set of covariates, yielding adjusted ORs (aORs). By changing the reference category to adolescents with co-occurring depression and AUD, we explored if this group differed from adolescents with only AUD or only depression, respectively.

Sex-stratified sub-analyses were conducted. Additional sub-analyses explored the prevalence of co-occurring depression and AUD in adulthood by combinations of depression and AUD in adolescence. Differences between the categories were tested using logistic regression, with co-occurring depression and AUD in adolescence as the reference category. Finally, sensitivity analyses were conducted excluding participants with only subsyndromal depression at baseline. The statistical analyses were conducted using Stata, version 17 [32].

## Results

### Sample characteristics

The study included 382 participants providing data at both baseline and the 15-year follow-up. Participants were predominantly female (79%). Overall, baseline characteristics were similar in the included sample and the full cohort they were drawn from (Table 1 and Supplementary material, Table S1).

**Table 1:** Characteristics of adolescents with depression and non-depressed controls at baseline and follow-up

	All	Depressed	Non-depressed controls	χ^2^	*p*-value
	% (n)	% (n)	% (n)		
Adolescence					
*Full cohort (n = 590-591)*					
AUD	13.2 (78)	17.4 (61)	7.1 (17)	**13.41**	**<0.001**
Drug use or abuse	0.3 (2)	0.6 (2)	0.0 (0)	1.38	0.240
Sex					
Female	78.0 (461)	78.3 (274)	77.6 (187)		
Male	22.0 (130)	21.7 (76)	22.4 (54)	0.04	0.842
Physical/sexual abuse	12.9 (76)	19.2 (67)	3.7 (9)	**30.38**	**<0.001**
Parental unemployment	10.8 (64)	12.9 (45)	7.9 (19)	3.66	0.056
Reduced income in the family	15.6 (92)	19.1 (67)	10.4 (25)	**8.35**	**0.004**
ADHD/defiant disorder/conduct disorder	20.6 (122)	30.3 (106)	6.6 (16)	**48.72**	**<0.001**
Major family conflicts	18.4 (109)	27.4 (96)	5.4 (13)	**46.07**	**<0.001**
*Included sampled (n = 367-382)*					
AUD	12.8 (49)	16.7 (38)	7.1 (11)	**7.66**	**0.006**
Drug use or abuse	0.5 (2)	0.9 (2)	0.0 (0)	1.37	0.241
Sex					
Female	79.3 (303)	79.3 (180)	79.4 (123)		
Male	20.7 (79)	20.7 (47)	20.6 (32)	0.00	0.989
Physical/sexual abuse	12.6 (48)	18.6 (42)	3.9 (6)	**18.08**	**<0.001**
Parental unemployment	10.0 (38)	11.9 (27)	7.1 (11)	2.37	0.124
Reduced income in the family	16.8 (64)	21.6 (49)	9.7 (15)	**9.37**	**0.002**
ADHD/defiant disorder/conduct disorder	19.9 (76)	29.5 (67)	5.8 (9)	**32.49**	**<0.001**
Major family conflicts	18.3 (70)	27.3 (62)	5.2 (8)	**30.20**	**<0.001**
Parental depression	32.7 (124)	37.5 (84)	25.8 (40)	**5.69**	**0.017**
Adulthood					
*Included sampled (n = 367-382)*					
Depression (M.I.N.I.)^a^	47.9 (183)	59.5 (135)	31.0 (48)	**29.99**	**<0.001**
Anxiety disorder (M.I.N.I.)^b^	35.1 (134)	44.1 (100)	21.9 (34)	**19.78**	**<0.001**
Suicidality (M.I.N.I.)^a^	27.0 (103)	35.2 (80)	14.8 (23)	**19.47**	**<0.001**
AUD (M.I.N.I.)^a^	10.7 (41)	13.2 (30)	7.1 (11)	3.60	0.058
AUD, past 12 months (M.I.N.I.)^b^	2.9 (11)	3.1 (7)	2.6 (4)	0.08	0.773
Hazardous drinking (AUDIT)^c^	16.1 (59)	19.4 (42)	11.3 (17)	**4.41**	**0.036**
Drug use disorders (M.I.N.I.)^a^	5.2 (20)	7.5 (17)	1.9 (3)	**5.73**	**0.017**
Drug use (DUDIT)^c^	1.6 (6)	2.8 (6)	0.0 (0)	**4.34**	**0.037**

*AUD* Alcohol Use Disorder

^a^Previous and ongoing episodes (M.I.N.I.)

^b^Age 19 to 30 for panic disorders; during the past 12 months for other anxiety disorders (M.I.N.I.)

^c^Current AUDIT score of ≥8 for men and ≥6 for women at follow-up

### Main analyses

The logistic regression analyses showed that adolescents with co-occurring depression and AUD were more likely than peers with neither of these conditions to encounter depression (aOR 5.33, 95% CI 2.22–12.83), anxiety disorders (aOR 4.05, 95% CI 1.77–9.27), suicidality (aOR 5.37, 95% CI 2.28-12.66), AUD (aOR 7.68, 95% CI 2.59–22.81), and hazardous drinking (aOR 4.21, 95% CI 1.56–11.35) in adulthood (Table 2). The adolescents with depression only were more likely than those with neither of the conditions to experience depression (aOR 2.66, 95% CI 1.62–4.35), anxiety disorders (aOR 2.08, 95% CI 1.23–3.52), suicidality (aOR 2.38, 95% CI 1.34–4.23), but not AUD and hazardous drinking. For adolescents with AUD only, there were no statistically significant associations with adult mental health outcomes.

**Table 2:** Associations of adolescent depression and AUD with adult mental health conditions (n = 349–382)

	Depression (age 19 to 30)^a^		Anxiety disorder (age around 30)^b^		Suicidality (age 19 to 30)^a^		AUD (age 19 to 30)^a^		Hazardous drinking (age around 30)^c^	
	Univariate^d^	Multivariate^e^	Univariate^d^	Multivariate^e^	Univariate^d^	Multivariate^e^	Univariate^d^	Multivariate^e^	Univariate^d^	Multivariate^e^
	OR (95% CI)	aOR (95% CI)	OR (95% CI)	aOR (95% CI)	OR (95% CI)	aOR (95% CI)	OR (95% CI)	aOR (95% CI)	OR (95% CI)	aOR (95% CI)
Condition in adolescence										
Neither depression nor AUD (ref.)	1.00	1.00	1.00	1.00	1.00	1.00	1.00	1.00	1.00	1.00
AUD but not depression	1.30 (0.36–4.66)	1.09 (0.28–4.15)	0.78 (0.16–3.78)	0.73 (0.15–3.64)	–	–	3.33 (0.62–17.78)	2.54 (0.42–15.13)	3.89 (0.90–16.75)	3.29 (0.72–15.06)
Depression but not AUD	**3.03** **(1.92–4.79)**	**2.66** **(1.62–4.35)**	**2.46** **(1.51–4.01)**	**2.08** **(1.23–3.52)**	**2.51** **(1.46–4.30)**	**2.38** **(1.34–4.23)**	1.48 (0.64–3.43)	1.35 (0.55–3.35)	1.59 (0.80–3.16)	1.37 (0.64–2.93)
Depression and AUD	**5.58** **(2.54–12.24)**	**5.33** **(2.22–12.83)**	**4.81** **(2.26–10.23)**	**4.05** **(1.77–9.27)**	**5.26** **(2.42–11.44)**	**5.37** **(2.28–12.66)**	**7.80** **(3.01–20.19)**	**7.68** **(2.59–22.81)**	**6.80** **(2.86–16.20)**	**4.21** **(1.56–11.35)**
Sex										
Female (ref.)	1.00	1.00	1.00	1.00	1.00	1.00	1.00	1.00	1.00	1.00
Male	**0.60** **(0.36–1.00)**	**0.48** **(0.27–0.85)**	**0.56** **(0.32–0.98)**	**0.50** **(0.27–0.92)**	**0.52** **(0.28–0.97)**	**0.46** **(0.23–0.91)**	1.69 (0.82–3.49)	1.46 (0.65–3.29)	1.83 (0.96–3.50)	1.74 (0.85–3.57)
Physical/sexual abuse	1.48 (0.80–2.72)	0.77 (0.38–1.58)	**2.03** **(1.10–3.73)**	1.60 (0.79–3.24)	**2.38** **(1.28–4.44)**	**2.28** **(1.10–4.70)**	1.50 (0.62**–**3.61)	1.78 (0.63**–**5.07)	0.95 (0.40**–**2.25)	0.58 (0.20**–**1.68)
Parental unemployment	1.99 (1.00–3.99)	2.03 (0.89–4.67)	0.84 (0.41–1.72)	0.51 (0.21–1.23)	0.96 (0.45–2.06)	0.74 (0.29–1.90)	1.66 (0.65–4.24)	1.85 (0.58–5.96)	1.12 (0.47–2.67)	0.55 (0.18–1.69)
Reduced income in the family	1.61 (0.94–2.78)	0.97 (0.50–1.87)	1.33 (0.77–2.31)	1.02 (0.52–1.99)	1.07 (0.59–1.95)	0.68 (0.32–1.44)	0.66 (0.25–1.76)	**0.24** **(0.07–0.84)**	1.32 (0.65–2.66)	1.37 (0.59–3.18)
ADHD/defiant disorder/conduct disorder	**2.03** **(1.21–3.39)**	1.29 (0.68–2.44)	**1.91** **(1.15–3.18)**	1.31 (0.70–2.45)	1.67 (0.98–2.85)	1.22 (0.62–2.38)	**3.00** **(1.51–5.96)**	1.83 (0.78–4.30)	**3.30** **(1.80–6.03)**	1.99 (0.93–4.24)
Major family conflicts	**2.45** **(1.42–4.21)**	1.61 (0.85–3.03)	**2.00** **(1.18–3.39)**	1.33 (0.72–2.46)	1.31 (0.74–2.30)	0.82 (0.42–1.61)	1.51 (0.70–3.25)	0.72 (0.28–1.83)	**2.04** **(1.08–3.86)**	1.37 (0.63–2.97)
Parental depression	**1.80** **(1.16–2.77)**	1.41 (0.88–2.26)	**1.63** **(1.05–2.54)**	1.45 (0.90–2.33)	1.24 (0.77–2.00)	1.15 (0.68–1.92)	**2.40** **(1.24–4.61)**	**2.42** **(1.17–5.00)**	1.62 (0.90–2.91)	1.37 (0.72–2.60)

*AUD* Alcohol Use Disorder

^a^Previous and ongoing episodes (M.I.N.I.)

^b^Age 19 to 30 for panic disorders; during the past 12 months for other anxiety disorders (M.I.N.I.)

^c^Current AUDIT score of ≥8 for men and ≥6 for women at follow-up

^d^Logistic regression, with statistically significant results in bold

^e^Logistic regression mutually adjusted for all variables, with statistically significant results in bold

When changing the reference category to adolescents with depression and AUD, some statistically significant differences were observed (Table 3). Compared to adolescents with both conditions, those with only depression had a significantly lower likelihood of experiencing suicidality (aOR 0.44, 95% CI 0.21–0.95), AUD (aOR 0.18, 95% CI 0.07–0.44) and hazardous drinking (aOR 0.33, 95% CI 0.14–0.77) in adulthood. Those with only AUD in adolescence had a significantly lower likelihood of experiencing depression (aOR 0.20, 95% CI 0.05–0.92) and anxiety disorders (aOR 0.18, 95% CI 0.03–0.99) in adulthood.

**Table 3:** Associations of adolescent depression and AUD with adult mental health conditions, with depression and AUD in adolescence as the reference category (n = 349–382)

	Depression (age 19 to 30)^a^		Anxiety disorder (age around 30)^b^		Suicidality (age 19 to 30)^a^		AUD (age 19 to 30)^a^		Hazardous drinking (age around 30)^c^	
	Univariate^d^	Multivariate^e^	Univariate^d^	Multivariate^e^	Univariate^d^	Multivariate^e^	Univariate^d^	Multivariate^e^	Univariate^d^	Multivariate^e^
	OR (95% CI)	aOR (95% CI)	OR (95% CI)	aOR (95% CI)	OR (95% CI)	aOR (95% CI)	OR (95% CI)	aOR (95% CI)	OR (95% CI)	aOR (95% CI)
Condition in adolescence										
Neither depression nor AUD	**0.18** **(0.08–0.39)**	**0.19** **(0.08–0.45)**	**0.21** **(0.10–0.44)**	**0.25** **(0.11–0.56)**	**0.19** **(0.09–0.41)**	**0.19** **(0.08–0.44)**	**0.13** **(0.05–0.33)**	**0.13** **(0.04–0.39)**	**0.15** **(0.06–0.35)**	**0.24** **(0.09–0.64)**
AUD but not depression	**0.23** **(0.06–0.96)**	**0.20** **(0.05–0.92)**	**0.16** **(0.03–0.85)**	**0.18** **(0.03–0.99)**	–	–	0.43 (0.08–2.28)	0.33 (0.05–2.04)	0.57 (0.13–2.58)	0.78 (0.16–3.87)
Depression but not AUD	0.54 (0.25–1.16)	0.50 (0.22–1.14)	0.51 (0.25–1.04)	0.51 (0.24–1.10)	**0.48** **(0.24–0.96)**	**0.44** **(0.21–0.95)**	**0.19** **(0.08–0.44)**	**0.18** **(0.07–0.44)**	**0.23** **(0.11–0.51)**	**0.33** **(0.14–0.77)**
Depression and AUD (ref.)	1.00	1.00	1.00	1.00	1.00	1.00	1.00	1.00	1.00	1.00

*AUD* Alcohol Use Disorder

^a^Previous and ongoing episodes (M.I.N.I.)

^b^Age 19 to 30 for panic disorders; during the past 12 months for other anxiety disorders (M.I.N.I.)

^c^Current AUDIT score of ≥8 for men and ≥6 for women at follow-up

^d^ Logistic regression, with statistically significant results in bold

^e^Logistic regression mutually adjusted for all variables, with statistically significant results in bold

The prevalence of depression, anxiety, suicidality, and AUD in adulthood by the presence of depression and AUD in adolescence is presented in the Supplementary material, Table S2.

### Additional analyses

**Fig 1:**
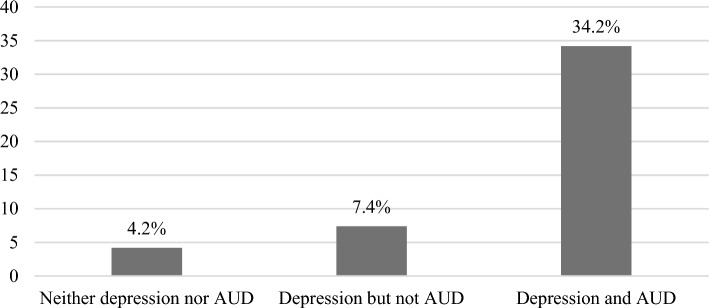
Proportions (%) of participants with co-occurring depression and alcohol use disorder (AUD) in adulthood (Y-axis) by conditions in adolescence (X-axis), with a statistically significant difference (p<0.001) between those with both depression and AUD in adolescence compared to those with neither of the conditions or only depression in adolescence (n = 371)

We further explored co-occurring depression and AUD in adulthood by different combinations of depression and AUD in adolescence (Fig 1). Among those with both depression and AUD in adolescence, 34.2% (n = 13) had both conditions also in adulthood, which differed significantly (p<0.001) from those with neither of the conditions (4.2%, n = 6) and those with depression only (7.4%, n = 14). Due to the small number of participants with only AUD in adolescence having co-occurring depression and AUD in adulthood, these results are not displayed.

Drug use disorder (M.I.N.I) and self-assessed drug use (DUDIT) in adulthood were reported by few participants (n = 6 and n = 20, respectively), and were therefore not included in the main analyses. Still, those having both depression and AUD in adolescence had a higher prevalence of adult drug use disorder (χ^2^ = 13.29, p = 0.004) and self-assessed drug use (χ^2^ = 38.13, p<0.001) in adulthood, compared with those with neither or only one of the conditions in adolescence (not presented in Table).

Sex-stratified analyses of adult outcomes suggested a similar overall pattern in both females and males (Supplementary material, Table S3), as did the sensitivity analyses excluding adolescents with subsyndromal depression (Tables S4 and S5).

## Discussion

This long-term follow-up study examined the association between co-occurring depression and AUD in adolescence and subsequent mental health outcomes in adulthood. To explore this relationship, we analysed longitudinal data from a diagnostically well-characterised and community-representative cohort of young individuals prospectively followed over a 15-year period.

Our findings suggest that individuals with co-occurring adolescent depression and AUD (i.e., dual diagnosis) had a particularly high likelihood of experiencing a negative mental health outcome, which was true for all outcomes examined. Notably, there was a statistically significant difference observed between the dual diagnosis group and those with adolescent depression only in terms of suicidality and AUD. In particular, the risk of continued co-occurring depression and AUD in adulthood was elevated. Individuals who experienced both adolescent depression and AUD also had a significantly higher likelihood of experiencing adult depression and anxiety disorder as compared to those with AUD alone. The observed associations between dual diagnosis and adverse outcomes persisted after adjusting for potential confounding factors, suggesting that the adverse impact on long-term outcomes is compounded by this comorbidity.

Few previous long-term follow-up studies of adolescent depression and AUD extend into early midlife. Our findings partially align with a New Zealand study by Boden & Foulds [23], using a similar design and follow-up period. Similar to their findings, we did not observe a significantly higher risk of adult depression in youth with a dual diagnosis compared to those with only depression. However, our results do suggest that youth with both depression and AUD face a significantly worse prognosis when considering a wider range of outcomes, including suicidality and co-occurring AUD.

What could account for the poorer prognosis of young people with co-occurring conditions in terms of mental health and AUD in adulthood? Previous studies have indicated that individuals with a dual diagnosis exhibit lower treatment adherence and poorer treatment outcomes compared to those with either depression or AUD alone [13, 23]. The comorbidity of depression and AUD may present challenges in achieving successful treatment outcomes [33, 34]. Consequently, this can hinder the individual’s ability to fully recover and lead to a more unfavourable long-term prognosis. Adolescents with ongoing AUD may also be underdiagnosed in the healthcare system [16], potentially leading to a lack of treatment for AUD altogether, further exacerbating the situation. Likewise, depression may be concealed by ongoing AUD, resulting in less effective treatment outcomes in addiction care. Suboptimal care may contribute to a prolonged duration of untreated or undertreated mental health issues, consequently increasing the risk of a negative prognosis.

Considering the cumulative impact on the brain of depression and AUD, it is also conceivable that the burden may be aggravated by the longitudinal course of this early-onset psychiatric comorbidity. Research has shown that AUD during adolescence can have neurocognitive consequences, including behavioural changes, attention deficits, impaired memory, and diminished verbal and visuospatial abilities [35]. Binge drinking, a prevalent pattern of alcohol consumption in Sweden, as well as abstinence and hangover symptoms, have been associated with the most pronounced neurological abnormalities during adolescence, particularly affecting the frontal, parietal, and temporal cortex [36]. Furthermore, a global study using brain imaging techniques has demonstrated widespread cortical changes in individuals with depression, suggesting that depression may dynamically influence brain structure, which may vary across different life stages [20].

Importantly, the social ramifications of depression [18] and AUD [19] are also likely to play a significant role in predicting an unfavourable prognosis. Previously reports based on ULADS suggest that depressed adolescents are at increased risk for a range of adverse social outcomes in adulthood, including labour market marginalisation [37], low income [38], and problems related to intimate relationships [39]. AUD may exacerbate these problems and compound the burden faced by individuals with co-occurring depression and AUD, further impeding their ability to thrive in various aspects of life.

### Clinical implications and future directions

The results of our work suggest dire outcomes for adolescents presenting with co-occurring depression and AUD. This poses the question of how best to clinically manage and treat this vulnerable group, especially within healthcare settings where current literature still reports modest treatment effects [34, 40]. Therefore, future research in this field would benefit from exploring whether timely rigorous treatment, combined (targeting both disorders) or in isolation can change the course of events. Rigorous treatment in this context would refer to combination pharmacotherapy, increased dosages, and possibly more psychotherapy sessions, especially in coordination with social services, where applicable. Furthermore, in view of the heterogeneity and complex pathophysiology of both AUD and depression, emphasis on personalised or patient-centred treatment is warranted [34]. Another area of research that this field could benefit from is an exploration of protective factors for adverse outcomes in this population, including social circumstances that might moderate or mediate the adverse effects of dual diagnosis. Such factors may be capitalised on and strengthened as part of the treatment care plan and possibly for preventive purposes.

### Strengths and limitations

A primary strength of this study lies in its long-term perspective, as participants were followed up after they transitioned into adulthood. The comparatively large community sample of adolescents increases the generalisability of the findings. In addition, all participants were assessed using structured diagnostic interviews and validated self-report questionnaires at baseline and at the 15-year follow-up, which undoubtedly is important from a clinical perspective.

The results should be interpreted in light of some limitations. Firstly, ULADS was not originally designed for the research questions addressed here. For instance, the group of adolescents with AUD but no depression during adolescence consisted of few individuals, which implies that any results based on this group must be interpreted with caution. Only individuals identified with depression or suicidality during the initial screening were invited for interviews. Those with only AUD were never called for an interview unless they were included in the control group. We still decided to include this subgroup in the analyses for transparency and to enable future synthesis with similar samples. Overall, the exploratory nature of this study underscores the necessity of independent replication of the findings. Secondly, approximately one-third of the eligible participants dropped out between the baseline and follow-up assessments. However, it is important to note that the dropout rate was similar between the depression group and the control group and there were no statistically significant differences in key variables between those who participated in the follow-up and those who did not. The high proportion of females in the sample reflects the higher prevalence of adolescent depression among females and was not related to attrition. Still, the small number of males prevents us from drawing firm conclusions related to sex-specific patterns. Thirdly, there were no measurement points between the ages of 16 and 30. As a result, some data obtained during the follow-up interview relied on retrospective recall, which may introduce a risk of recall bias [41]. In addition, some outcome measures focused only on the current state at follow-up. Fourthly, although associations were adjusted for a range of child and adolescent characteristics, residual confounding cannot be ruled out. Likewise, we did not investigate the mediating role of psychosocial circumstances in young adulthood. Finally, findings should be interpreted bearing the historical context of this cohort in mind. Global societal trends (e.g., environmental, social, economic, political, or technological change) in combination with declining mental health among youth over the last two decades make the generalisability of our findings uncertain [42]. In addition, alcohol consumption among adolescents has decreased in past decades [43]. Still, we believe the overall pattern of results can be generalised across generations.

## Conclusion

The findings of our study suggest that adolescents diagnosed with both depression and AUD are likely to experience an unfavourable long-term mental health prognosis compared to healthy controls and adolescents with depression or AUD only. Recognising teenagers with concurrent depression and AUD as a particularly vulnerable population holds clinical significance, as it allows for the possibility of early identification and prioritisation of treatment and resources. Indeed, several treatment studies suggest effects in a shorter timeframe. Addressing the specific needs of this group has the potential to ameliorate their long-term mental health prognosis and overall well-being.

## Supplementary Information

Below is the link to the electronic supplementary material.

Supplementary file1 (DOCX 41 KB)

## Data Availability

The data used for the current study are available from Uppsala University but restrictions apply to the availability of these data, and are therefore not publicly available. Data are however available from the Principal Investigator Dr. Ulf Jonsson (ulf.jonsson@ki.se) upon reasonable request and with permission of Uppsala University and ethical approval from the Swedish Ethical Review Authority.
