# Supplementary material for: Adult mental health outcomes of adolescent depression and co-occurring alcohol use disorder: a longitudinal cohort study
Source: Eur Child Adolesc Psychiatry. 2024 Oct 29;34(5):1649–59. doi: 10.1007/s00787-024-02596-3 (PMC12122575; doi:10.1007/s00787-024-02596-3)
Supplement: Supplementary file 1 — Supplementary file1 (DOCX 41 KB) [file 787_2024_2596_MOESM1_ESM.docx]

Supplementary material

**Table S1** Associations of conditions in adolescence measured at baseline in adolescence and participation in the follow-up (n=590)

|  | Participation in the follow-up |
| --- | --- |
|  | Multivariate |
|  | aOR  (95% CI) |
| *Conditions in adolescence* |  |
| Depression | 1.05  (0.72-1.53) |
| AUD | 0.92  (0.55-1.53) |
| Sex |  |
| Female (ref.) | 1.00 |
| Male | 0.83  (0.55-1.24) |
| Physical/sexual abuse | 0.91  (0.53-1.57) |
| Parental unemployment | 0.66  (0.37-1.19) |
| Reduced income in the family | 1.58  (0.92-2.70) |
| ADHD/defiant disorder/conduct disorder | 0.89  (0.56-1.41) |
| Major family conflicts | 1.00  (0.62-1.62) |

**Table S2** Adult mental health conditions by the presence of depression and AUD in adolescence (n=367-382)

|  | Depression (age 19 to 30)^a^ | Anxiety disorder (age around 30)^b^ | Suicidality (age 19 to 30)^a^ | AUD (age 19 to 30)^a^ | Hazardous drinking  (age around 30)^c^ |
| --- | --- | --- | --- | --- | --- |
|  | % (n) | % (n) | % (n) | % (n) | % (n) |
| Condition in adolescence |  |  |  |  |  |
| Neither depression nor AUD (n=144) | 30.6 (44) | 22.2 (32) | 16.0 (23) | 6.3 (9) | 9.9 (14) |
| AUD but not depression (n=11) | 36.4 (4) | 18.2 (2) | 0.0 (0) | 18.2 (2) | 30.0 (3) |
| Depression but not AUD (n=189) | 57.1 (108) | 41.3 (78) | 32.3 (61) | 9.0 (17) | 14.9 (27) |
| Depression and AUD (n=38) | 71.1 (27) | 57.9 (22) | 50.0 (19) | 34.2 (13) | 42.9 (15) |
|  |  |  |  |  |  |
| χ^2^ | **32.58** | **23.70** | **25.84** | **26.12** | **24.17** |
| p-value^d^ | **<0.001** | **<0.001** | **<0.001** | **<0.001** | **<0.001** |

*AUD* Alcohol Use Disorder

^a^Previous and ongoing episodes (M.I.N.I.)

^b^Age 19 to 30 for panic disorders; during the past 12 months for other anxiety disorders (M.I.N.I.)

^c^Current AUDIT score of ≥8 for men and ≥6 for women at follow-up

^d^Differences between categories, with statistically significant results in bold

**Table S3** Adult mental health conditions by the presence of depression and AUD in adolescence, stratified by sex

|  | Depression (age 19 to 30)^a^ | Anxiety disorder (age around 30)^b^ | Suicidality (age 19 to 30)^a^ | AUD (age 19 to 30)^a^ | Hazardous drinking  (age around 30)^c^ |
| --- | --- | --- | --- | --- | --- |
| **Women (n=299-303)** | % (n) | % (n) | % (n) | % (n) | % (n) |
| Condition in adolescence |  |  |  |  |  |
| Neither depression nor AUD | 33.0 (38) | 24.4 (28) | 16.5 (19) | 5.2 (6) | 10.5 (12) |
| AUD but not depression | Not shown^d^ | Not shown^d^ | Not shown^d^ | Not shown^d^ | Not shown^d^ |
| Depression but not AUD | 60.8 (93) | 43.1 (66) | 36.0 (55) | 8.5 (13) | 12.7 (19) |
| Depression and AUD | 70.4 (19) | 66.7 (18) | 55.6 (15) | 37.0 (10) | 37.0 (10) |
| χ^2^ | **25.30** | **20.87** | **24.59** | **27.10** | **13.72** |
| p-value^e^ | **<0.001** | **<0.001** | **<0.001** | **<0.001** | **0.003** |
|  |  |  |  |  |  |
| **Men (n=68-79)** | % (n) | % (n) | % (n) | % (n) | % (n) |
| Condition in adolescence |  |  |  |  |  |
| Neither depression nor AUD | 20.7 (6) | 13.8 (4) | 13.8 (4) | 10.3 (3) | 7.4 (2) |
| AUD but not depression | Not shown^d^ | Not shown^d^ | Not shown^d^ | Not shown^d^ | Not shown^d^ |
| Depression but not AUD | 41.7 (15) | 33.3 (12) | 16.7 (6) | 11.1 (4) | 25.8 (8) |
| Depression and AUD | 72.7 (8) | 36.4 (4) | 36.4 (4) | 27.3 (3) | 62.5 (5) |
| χ^2^ | **9.55** | 4.99 | 3.60 | **8.41** | **11.52** |
| p-value | **0.023** | 0.173 | 0.308 | **0.038** | **0.009** |

*AUD* Alcohol Use Disorder

^a^Previous and ongoing episodes (M.I.N.I.)

^b^Age 19 to 30 for panic disorders; during the past 12 months for other anxiety disorders (M.I.N.I.)

^c^Current AUDIT score of ≥8 for men and ≥6 for women at follow-up

^d^Few participants; data not shown

^e^Differences between categories, with statistically significant results in bold

**Table S4** Associations of depression and AUD in adolescence with adult mental health conditions, excluding cases with subsyndromal depression at baseline (n=315-342)

|  | Depression (age 19 to 30)^a^ | | Anxiety disorder (age around 30)^b^ | | Suicidality (age 19 to 30)^a^ | | AUD (age 19 to 30)^a^ | | Hazardous drinking  (age around 30)^c^ | |
| --- | --- | --- | --- | --- | --- | --- | --- | --- | --- | --- |
|  | Univariate^d^ | Multivariate^e^ | Univariate^d^ | Multivariate^e^ | Univariate^d^ | Multivariate^e^ | Univariate^d^ | Multivariate^e^ | Univariate^d^ | Multivariate^e^ |
|  | OR  (95% CI) | aOR  (95% CI) | OR  (95% CI) | aOR  (95% CI) | OR  (95% CI) | aOR  (95% CI) | OR  (95% CI) | aOR  (95% CI) | OR  (95% CI) | aOR  (95% CI) |
| Condition in adolescence |  |  |  |  |  |  |  |  |  |  |
| Neither depression nor AUD (ref.) | 1.00 | 1.00 | 1.00 | 1.00 | 1.00 | 1.00 | 1.00 | 1.00 | 1.00 | 1.00 |
| AUD but not depression | 1.30  (0.36-4.66) | 1.07  (0.28-4.13) | 0.78  (0.16-3.78) | 0.76  (0.15-3.78) | - | - | 3.33  (0.62-17.78) | 2.74  (0.47-16.13) | 3.89  (0.90-16.75) | 3.26  (0.72-14.75) |
| Depression but not AUD | **3.87**  **(2.39-6.27)** | **3.30**  **(1.94-5.62)** | **3.07**  **(1.86-5.09)** | **2.68**  **(1.55-4.64)** | **2.84**  **(1.63-4.95)** | **2.70**  **(1.48-4.93)** | 1.62  (0.69-3.82) | 1.54  (0.60-3.95) | 1.57  (0.77-3.21) | 1.26  (0.56-2.83) |
| Depression and AUD | **7.10**  **(2.97-16.98)** | **7.43**  **(2.76-19.97)** | **6.13**  **(2.72-13.78)** | **5.40**  **(2.24-13.05)** | **4.95**  **(2.19-11.19)** | **5.11**  **(2.08-12.56)** | **8.57**  **(3.22-22.81)** | **9.85**  **(3.18-30.55)** | **7.06**  **(2.90-17.18)** | **4.55**  **(1.66-12.50)** |
| Sex |  |  |  |  |  |  |  |  |  |  |
| Female (ref.) | 1.00 | 1.00 | 1.00 | 1.00 | 1.00 | 1.00 | 1.00 | 1.00 | 1.00 | 1.00 |
| Male | **0.53**  **(0.31-0.92)** | **0.45**  **(0.24-0.83)** | 0.56  (0.31-1.01) | 0.53  (0.28-1.00) | 0.52  (0.27-1.03) | 0.52  (0.25-1.06) | 1.55  (0.71-3.37) | 1.57  (0.66-3.71) | 1.23  (0.59-2.56) | 1.20  (0.54-2.68) |
| Physical/sexual abuse | 1.66  (0.86-3.18) | 0.87  (0.40-1.87) | **2.15**  **(1.13-4.09)** | 1.59  (0.76-3.32) | **2.40**  **(1.24-4.62)** | **2.13**  **(1.00-4.51)** | 1.67  (0.69-4.08) | 2.28  (0.80-6.51) | 1.09  (0.46-2.61) | 0.73  (0.25-2.11) |
| Parental unemployment | **2.09**  **(1.00-4.34)** | 2.34  (0.96-5.69) | 0.87  (0.42-1.82) | 0.57  (0.23-1.38) | 1.08  (0.50-2.34) | 0.91  (0.35-2.32) | 1.78  (0.69-4.61) | 2.31  (0.71-7.48) | 1.22  (0.51-2.93) | 0.59  (0.19-1.85) |
| Reduced income in the family | 1.58  (0.90-2.77) | 0.84  (0.42-1.68) | 1.32  (0.75-2.32) | 0.97  (0.49-1.93) | 1.04  (0.56-1.93) | 0.65  (0.30-1.38) | 0.67  (0.25-1.80) | **0.22**  **(0.06-0.81)** | 1.41  (0.69-2.88) | 1.39  (0.59-3.25) |
| ADHD/defiant disorder/conduct disorder | **2.02**  **(1.16-3.52)** | 1.08  (0.54-2.18) | **1.94**  **(1.12-3.33)** | 1.19  (0.61-2.31) | 1.57  (0.89-2.80) | 1.03  (0.51-2.08) | **2.46**  **(1.18-5.12)** | 1.33  (0.54-3.28) | **2.83**  **(1.49-5.39)** | 1.56  (0.70-3.51) |
| Major family conflicts | **2.81**  **(1.56-5.06)** | 1.82  (0.90-3.67) | **2.00**  **(1.15-3.47)** | 1.23  (0.64-2.37) | 1.44  (0.80-2.59) | 0.90  (0.44-1.83) | 1.44  (0.64-3.21) | 0.73  (0.28-1.91) | **2.19**  **(1.13-4.26**) | 1.55  (0.69-3.47) |
| Parental depression | **1.79**  **(1.13-2.82)** | 1.34  (0.81-2.22) | 1.48  (0.93-2.34) | 1.24  (0.75-2.05) | 1.27  (0.77-2.08) | 1.12  (0.65-1.92) | **2.38**  **(1.21-4.71)** | **2.42**  **(1.14-5.12)** | 1.75  (0.95-3.23) | 1.48  (0.76-2.88) |

^a^Previous and ongoing episodes (M.I.N.I.)

^b^Age 19 to 30 for panic disorders; during the past 12 months for other anxiety disorders (M.I.N.I.)

^c^Current AUDIT score of ≥8 for men and ≥6 for women at follow-up

^d^ Logistic regression, with statistically significant results in bold

^e^Logistic regression mutually adjusted for all variables, with statistically significant results in bold

**Table S5** Associations of depression and AUD in adolescence with adult mental health conditions, with depression and AUD in adolescence as the reference category excluding cases with subsyndromal depression at baseline (n=315-342)

|  | Depression (age 19 to 30)^a^ | | Anxiety disorder (age around 30)^b^ | | Suicidality (age 19 to 30)^a^ | | AUD (age 19 to 30)^a^ | | Hazardous drinking  (age around 30)^c^ | |
| --- | --- | --- | --- | --- | --- | --- | --- | --- | --- | --- |
|  | Univariate^d^ | Multivariate^e^ | Univariate^d^ | Multivariate^e^ | Univariate^d^ | Multivariate^e^ | Univariate^d^ | Multivariate^e^ | Univariate^d^ | Multivariate^e^ |
|  | OR  (95% CI) | aOR  (95% CI) | OR  (95% CI) | aOR  (95% CI) | OR  (95% CI) | aOR  (95% CI) | OR  (95% CI) | aOR  (95% CI) | OR  (95% CI) | aOR  (95% CI) |
| Condition in adolescence |  |  |  |  |  |  |  |  |  |  |
| Neither depression nor AUD | **0.14**  **(0.06-0.34)** | **0.13**  **(0.05-0.36)** | **0.16**  **(0.07-0.37)** | **0.19**  **(0.08-0.45)** | **0.20**  **(0.09-0.46)** | **0.20**  **(0.08-0.48)** | **0.12**  **(0.04-0.31)** | **0.10**  **(0.03-0.31)** | **0.14**  **(0.06-0.35)** | **0.22**  **(0.08-0.60)** |
| AUD but not depression | **0.18**  **(0.04-0.79)** | **0.14**  **(0.03-0.70)** | **0.13**  **(0.02-0.69)** | **0.14**  **(0.02-0.79)** | - | - | 0.39  (0.07-2.10) | 0.28  (0.05-1.72) | 0.55  (0.12-2.52) | 0.72  (0.15-3.53) |
| Depression but not AUD | 0.54  (0.23-1.29) | 0.44  (0.17-1.16) | 0.50  (0.23-1.09) | 0.50  (0.22-1.13) | 0.57  (0.27-1.23) | 0.53  (0.24-1.19) | **0.19**  **(0.08-0.46)** | **0.16**  **(0.06-0.41)** | **0.22**  **(0.10-0.51)** | **0.28**  **(0.11-0.68)** |
| Depression and AUD (ref.) | 1.00 | 1.00 | 1.00 | 1.00 | 1.00 | 1.00 | 1.00 | 1.00 | 1.00 | 1.00 |

*AUD* Alcohol Use Disorder

^a^Previous and ongoing episodes (M.I.N.I.)

^b^Age 19 to 30 for panic disorders; during the past 12 months for other anxiety disorders (M.I.N.I.)

^c^Current AUDIT score of ≥8 for men and ≥6 for women at follow-up

^d^ Logistic regression, with statistically significant results in bold

^e^Logistic regression mutually adjusted for all variables, with statistically significant results in bold
